# Quality management of eLearning for medical education: current situation and outlook

**DOI:** 10.3205/zma000962

**Published:** 2015-05-13

**Authors:** Jasmin Abrusch, Jörg Marienhagen, Anja Böckers, Susanne Gerhardt-Szép

**Affiliations:** 1 Goethe University Frankfurt am Main, Carolinum Dental University-Institute gGmbH, Department of Operative Dentistry, Frankfurt, Germany; 2 Clinic of University Regensburg, Medical Faculty, Department of Nuclear Medicine, Germany; 3 University of Ulm, Medical Faculty, Institute of Anantomy and Cell Biology, Germany

**Keywords:** education, eLearning, quality, quality assurance, quality management

## Abstract

**Introduction:** In 2008, the German Council of Science had advised universities to establish a quality management system (QMS) that conforms to international standards. The system was to be implemented within 5 years, i.e., until 2014 at the latest. The aim of the present study was to determine whether a QMS suitable for electronic learning (eLearning) domain of medical education to be used across Germany has meanwhile been identified.

**Methods: **We approached all medical universities in Germany (n=35), using an anonymous questionnaire (8 domains, 50 items).

**Results:** Our results (response rate 46.3%) indicated very reluctant application of QMS in eLearning and a major information deficit at the various institutions.

**Conclusions:** Authors conclude that under the limitations of this study there seems to be a considerable need to improve the current knowledge on QMS for eLearning, and that clear guidelines and standards for their implementation should be further defined.

## Zusammenfassung

**Einleitung:** Der Wissenschaftsrat empfahl 2008 den Universitäten innerhalb der nächsten 5 Jahre, d. h. bis spätestens 2014, ein Qualitätsmanagementsystem (QMS), das internationalen Maßstäben entspricht, zu etablieren. Ziel der vorliegenden Studie war es, zu evaluieren, ob es derzeit ein geeignetes QMS für das elektronische Lernen (eLearning) gibt, das speziell im Fach Humanmedizin deutschlandweit eingesetzt werden kann.

**Methoden:** Im Rahmen einer Umfrage wurden mittels eines anonymisierten Fragebogens (8 Domänen, 50 Items) alle Universitäten (n=35) des Fachbereichs Medizin in Deutschland evaluiert.

**Ergebnisse: **Die Ergebnisse (46,3% Rücklaufquote) zeigen einen nur zögerlichen Einsatz von QMS für eLearning und dass vor Ort ein großes Informationsdefizit herrscht.

**Schlussfolgerung:** Unter Berücksichtigung der Limitationen dieser Studie kann zusammenfassend festgehalten werden, dass erheblicher Bedarf zu bestehen scheint, das existierende Informationsdefizit für QMS eLearning zu mindern, sowie zukünftig genaue Richtlinien und Standards zur Umsetzung zu definieren.

## Introduction

Electronic learning (eLearning) is increasingly used at universities and is expected to gain even more importance in the future. Universities have a legal obligation to evaluate the quality and effectiveness of their teaching [http://www.gesetze-im-internet.de/hrg/BJNR001850976.html cited 2014 September 10]. While in the past, this obligation concerned only classroom teaching, it now has to be extended to the eLearning domain. Because clear guidelines are still missing, the process of quality management is impeded. In their recommendation of July 04, 2008, the German Council of Science had stated that within a period of approximately three to five years, universities should establish a quality management system (QMS) that meets international standards [1]. Furthermore it recommended the implementation of reliable tools to evaluate the quality of teaching [1]. 

Thus, universities have to decide which type of QMS they wish to adopt and integrate. The aim of the present study was to assess the current situation regarding the use of QMS in eLearning by sending an anonymous questionnaire to all German medical universities and some related institutions that use eLearning tools. Our working hypothesis was as follows: „Although early initiatives of quality management for eLearning in medical education do exist, adoption and realisation of QMS at the universities are barely apparent. Universities lack knowledge of these systems, and guidelines and standards for their implementation are missing“.

## Material and methods

### Study participants

The study population consisted of all German medical schools (n=35) as well as some non-university institutions or departments other than medical schools (n=6) that were known to use a QMS for eLearning or to take an interest in this matter. Institutions not located in Germany were excluded. Names of individuals responsible for quality management (specifically for eLearning, if available) at the selected universities were retrieved from the institutions‘ homepages. In addition, we searched for addresses of deaneries and administrative offices. To ensure a high rate of returned questionnaires, we informed the potential participants about the study by e-mail before sending out the questionnaires. Content and scope of the study were explained, and participating institutions were asked to provide the e-mail address of a contact person to whom the questionnaire could be sent. Contact persons without an e-mail address were contacted by telephone to request the current details for correspondence. Content and scope of the study were detailed again when sending out the final questionnaire, and instructions on the completion of both the paper&pencil version (provided as a PDF attachment for printout on paper and return by mail) and the online version (including the TAN number of the Education Survey Automation Suite [EvaSys] platform). Twenty-one days after dispatch of the forms, a first e-mail reminder was sent out, with a second and final one released after another 21 days. In both e-mails, the selected institutions were asked again to participate in the study, emphasising the value and importance of their individual replies. 

#### Questionnaire

The questionnaire contained 50 items in 8 domains (see attachment: ). It comprised “closed” questions providing a choice of answers, open questions with blanks for free text entries, and questions on personal opinion using a 5-point scale (1=totally agree, 2=agree, 3=neutral, 4=disagree, 5=totally disagree). 

The eight domains included questions regarding

1. general informations about the institution (6 Items),
2. general informations about QMS at the institution (11 items),
3. decision making (11 items),
4. satisfaction, clarity (5 items),
5. time factors (4 items),
6. cost factors (4 items),
7. headcount and responsabilities (6 items) and
8. general comments (3 items).

The content of our questionnaire was based on two earlier surveys, i.e., a study performed by the European Quality Observatory (EQO) entitled “Use and prevalence of quality approaches for eLearning in Europe” [2], and a study on quality management in private medical practices conducted by the German Public Health Foundation (GPHF) in 2008 [3]. We adapted the questions asked by GPHF (e.g., whether institutions have considered quality management at all, and which types of QMS they know) to eLearning and the current test population. The study by the EQO was used to formulate questions on the degree of popularity of various approaches to managing quality [2]. 

The QMS for eLearning most commonly discussed in the literature were listed as options to tick in the questionnaire. They comprised CEL, DIN PAS 1032-1, Q.E.D, QSel, TUD eLearning-Label, and WebKolleg NRW. The specific features of these QMS are as follows:

1. CEL: The CEL (certification of eLearning) evaluates and develops quality on the meso level (university degree courses, educational programmes). The system is concerned with quality assessment of educational programmes supported by eLearning. Thus, the CEL assesses the complete training module rather than the product itself. It is planned to establish CEL for the entire European eLearning sector.
2. DIN-PAS 1032-1: This QMS concerns primary and continued education with a specific focus placed on eLearning. It was developed by the working group named “Quality in eLearning” of the German Institute for Standardization (DIN; Deutsches Institut für Normung e.V.; PAS = Publicly Available Specifications). The system aims to elaborate processes for planning, developing, conducting, and evaluating educational programmes and facilities, particularly those supported by eLearning.
3. Q.E.D.: The Q.E.D. (quality initiative eLearning in Germany; Qualitätsinitiative eLearning in Deutschland) deals with primary and continued education with a special focus placed on eLearning. The initiative’s primary objective is to improve the quality of work process-oriented eLearning in Germany, based on the development and implementation of reference models and quality standards.
4. QSel: The QSeL (seal of quality eLearning; Qualitätssiegel eLearning) aims to improve the quality of organisations concerned with primary und continued education, especially those using eLearning and blended learning. The seal serves to document and certify practical application of the quality models DIN- PAS 1032–1 and ISO/IEC 19796–1. In this way, the seal complements existing approaches, concepts, and processes of quality management in the area of eLearning. The seal does not attempt to evaluate the products themselves but rather serves as an instrument to assess process-oriented goals and their degree of achievement.
5. TUD eLearning-Label: The TUD (Technical University of Darmstadt) eLearning-Label evaluates eLearning activities. The system serves as the quality standard to ensure educational and didactic quality of information/communication technology used as part of university course curricula. Educational modules are awarded the label if the criterion of improved teaching benefits is fulfilled. At present, no TUD eLearning-Labels are awarded.
6. WebKolleg NRW: WebKolleg NRW (Nordrhein-Westfalen) assesses educational programmes, especially those involving eLearning, on the basis of more than 50 criteria relating to contents, methodological-didactic aspects, and technical features. Approval is based on criteria developed by WebKolleg NRW themselves, and no certificate or seal is awarded. WebKolleg NRW provides a procedure for authorizing educational modules for the continued-education portal of Nordrhein-Westfalen.

In the questionnaire, a space was added for entry of any system that was not listed. The final questionnaire was developed in a three-step procedure. A team of nine employees of the dental clinic of the Goethe University, Frankfurt am Main (a master of medical education, a QMS delegate, and seven members of the medical education staff) was responsible for evaluating the appropriateness of the questionnaire on three occasions (March, July, and August 2010), using the ‘thinking aloud’ method [4]. The team members were asked to review the existing version of the questionnaire and were instructed to clearly voice their comments. These were immediately noted and documented by the study leader in a circulating process. The method requires that the team members “think aloud” while performing a specific task (e.g., review of the questionnaire in our case). The team members were asked to pronounce everything that occurred to them, as well as what they were doing, feeling, and looking at while performing the assigned task. This method is advantageous because any misinterpretations of concepts applied in the system and their reasons become readily apparent. 

#### Data collection and analysis

Data collection lasted from September 07, 2010, to March 31, 2011. Data of completed questionnaires were entered into EvaSys for metric evaluation.

## Results

### Return rates and integration

Overall, 19 of the 41 questionnaires sent out were returned. Of the 35 questionnaires sent to German medical universities, 16 were resubmitted (i.e., Aachen, Berlin, Düsseldorf, Essen, Freiburg, Göttingen, Greifswald, Halle, Hamburg, Hannover, Heidelberg, Jena, Kiel, Leipzig, Lübeck, and Ulm). Of the 6 questionnaires sent to non-university institutions or departments other than medical, three (Technical University of Darmstadt, Wilhelm Büchner University of Darmstadt, and the Central Quality Assurance Section of the Johannes Gutenberg University im Mainz) were resubmitted. Of the 19 questionnaires returned, only three were answered completely. There existed 6 items partially not being answered by all these remaining 16 institutions: numbers 2.4 (answer: “We could not yet decide on a QMS”), 3.1 (answers: “cost effectiveness / reduction of costs” and “streamlining of processes”), 3.3 (answers: “cost factor”, “recommendation”, “competition”, “time factor” and “competent service provider”), 3.6 (answers: “not satisfied with the service provider” and “individual adaptation to specific requirements not or hardly possible”), 4.4 (answers: “economic efficiency” and “improved learning outcomes”) and 7.5 (trained QM delegate). Three of them were part of the domain “decision making” the others of the domains “general question””, “satisfaction/clarity”, and “headcount/responsabilties”.

The study revealed that 14 institutions had adopted a QMS. Nine institutions reported a general QMS to be available for the entire institution. Only three participating institutions stated the use of a QMS specifically designed for eLearning. Two of them were medical universities. However, six institutions indicated that a general QMS for teaching is available. In nine cases where no QMS for eLearning was established, this was justified by a lack of information about QMS for eLearning. In two cases, the reasons given were “no time” and “eLearning is not or hardly used in teaching”, and in one case, “costs too high” or “no need/no interes” was stated. Further reasons listed were, for instance: “eLearning is still mainly governed by the individual clinics/departments; barely any inter-department organisation present”, “Introduction is planned by the central university administration: general quality management and quality management of teaching”, “We have a student evaluation system, but no QMS in the strictest sense”, or “Our general QMS also covers eLearning programmes, and therefore no separate system is needed”. The data received including parameters such as the degree of popularity of QMS for eLearning, effectiveness, time and costs for implementing such QMS can not be stated as representative, because of the minimal response rate of the questionnaires.

## Discussion

Bearing in mind our working hypothesis the achieved results show that only a few medical universities in Germany have introduced a QMS for general teaching and learning so far. The minimum number of implemented QMS for eLearning expecially results in a hard limitation of the study interpretation. Also the response rate of the study and the fact that of the 19 questionnaires returned, only three were answered completely, must be critically mentioned during the discussion of our results. Furthermore extrapolation of the findings to other disciplines and countries is not possible. However it must be stated, that six from the 50 items were generally not answered completely by the 16 remaining institutions. Mostly questions about decision making themes were dropped out, for example as reasons, why the eLearning QMS wouldn`t be choosen again with the answers “not satisfied with the service provider” and “individual adaptation to specific requirements not or hardly possible”. In the domain “satisfaction and clarity” the answers to the question “Which positive effects have resulted from the implementation of QMS for eLearning?” with “economic efficiency” and “improved learning outcomes” were never ticked. In the domain “headcount and responsabilities” the answer of the question “Who is responsible for the QMS for eLearning?” with “trained QM delegate” was also never marked. These facts must be considered with regard to the current situation. Perhaps the omission of possible answers reflects the problem of the respondents. Further research should clarify these relationships.

Our study documents however that general QMS is well established in medical German university hospitals, while suitable QMS for eLearning are barely available.

Current literature indicates that specific QMS for eLearning do exist, but information on their implementation at universities is limited. It is apparent that establishing such systems at universities is associated with various difficulties [http://www.hamburg.de/contentblob/4014946/data/gesetzesentwurf-zur-weiterentwicklung-des-hochschulrechts.pdf, cited 2014 September 10], [5], [6], [7]. Universities have to decide whether to use an internal or external QMS and whether the system should be product- or process-oriented. Moreover, decisions with respect to quality criteria (specifically developed versus predefined) and the choice of a controller (external versus in-house) have to be made. Finally, questions concerning the duration of the implementation process and availability of budget must be addressed. 

According to Bremer [5], the level of quality management at universities specially in German-speaking countries is rather heterogeneous at present. While some universities, e.g., the University of St. Gallen, Switzerland, the Technical University Darmstadt, and the Goethe University, Frankfurt am Main, have initiated quality assessments of their eLearning products and settings, others are mostly concerned with establishing rather than monitoring eLearning modules at their institution [5]. Thus, evaluation of eLearning tools at universities is still in its early stages [7], and QMS for eLearning are rather rare [2]. Although it is essential to establish evaluation processes within the framework of quality management [http://www.hamburg.de/contentblob/4014946/data/gesetzesentwurf-zur-weiterentwicklung-des-hochschulrechts.pdf, cited 2014 September 10], integration of complete QMS at universities is only just beginning [6], and it remains uncertain how such systems will perform at individual institutions. 

Validated instruments for the comprehensive assessment of QMS for eLearning in medical training are not yet available. The studies (EQO and GPHF) utilized in our investigation were used as a rough guidance only because they were unrelated to medical contents or referred to general QMS rather than specific QMS for eLearning [2], [3]. 

As reported in the literature, most QMS for eLearning were completed after 2002. For example, DIN PAS1032-1:2004 [8], DIN-PAS1037-2004 [8], and TUD eLearning Label [9] were all completed in 2004, while Q.E.D. [8] was introduced in 2006. WebKolleg NRW [http://www.webkolleg.nrw.de, cited 2014 September 10] became available in 2003. The positive effects expected to result from the implementation of a QMS for eLearning, such as improved quality of teaching, larger selection of eLearning programmes, enhanced competitiveness, lower error rate, streamlined processes, and improved learning outcomes, are stated in literature [8]. Information on quality management of university teaching on an international level has emerged from surveys conducted by Fredekind et al. [10] and Ruiz et al. [11]. The authors suggest peer-reviewed models for eLearning programmes, pointing out that eLearning is increasingly used in clinical teaching but is not yet subject to peer-review. 

## Degree of popularity

Nine institutions indicated that the reason for not having introduced a QMS for eLearning was the lack of relevant information. This agrees well with other studies, although the popularity level for general QMS among medical doctors amounted to 73% for the system DIN EN ISO 9000 ff. and to 28.4% for EFQM (the excellence model of the European Foundation for Quality Management) [3]. A variation of the EFQM, namely the EFQUEL (European Foundation for Quality in e-Learning) certifies additionally to medical universities also non-medical institutions like DOBA (Maribor Faculty of Applied Business and Social Studies in Slovenia), Moscow university of Industry and Finance in Russia, School of Humanities at the University of Aegean in Greece. In the literature, specific training and the appointment of an external quality manager have frequently been named as possible solutions to increase the popularity level. According to Wellems [12], it may be helpful to involve an external expert whose acceptance by the staff is expected to be higher than that of an internal expert. In this way, costs in connection with internal quality management training may be reduced [12]. 

In the literature, the time needed to establish a QMS in general education, e.g., learner-oriented quality improvement in advanced training (LQW), was indicated as 13 months [http://www.elearning-mv.de/wp-content/uploads/2011/12/EmpfehlungenZertifizierungE-Learning-in-MV.pdf, cited 2014 September 10], while the Central Office for Distance Learning (ZFU) cited 90 days [http://www.zfu.de, cited 2014 September 10], and the “Gütesiegelverbund Weiterbildung” reported 12 months [http://www.guetesiegelverbund.de, cited 2014 September 10]. For WebKolleg NRW, the time needed to set up the system was documented as one day [http://www.webkolleg.nrw.de, cited 2014 September 10]. Using the GPHF-study as an example, the time required for QMS implementation amounted to 6 h per week for the staff, and 4.5 h per week for the owner of the private practice [3]. Quality competitions, such as the EQA (European Quality Award), require extensive preparation time lasting several years [13]. Similarly, extensive preparation and consultation time is required for DIN-PAS 1032-1 that comprises 693 quality criteria [8]. 

However, it is not always necessary to set up a separate QMS for eLearning; in certain cases, integration into a pre-existing QMS may be possible [14]. In the literature, the saving potential of a QMS is indicated mostly in terms of re-usability (standards) [http://www.elearning-mv.de/wp-content/uploads/2011/12/EmpfehlungenZertifizierungE-Learning-in-MV.pdf, cited 2014 September 10]. 

Regarding the costs, only limited information on the set-up and maintenance of QMS for eLearning is available. The literature indicates that universities receive variable financial support. An example for only modest support was described by Hendricson et al. [15], who assessed the realisation of eLearning curricula at medical universities in North-America. The authors concluded from their study that only few faculties obtained financial support for the implementation of a QMS. Fredekind et al. [10] reported also an example where support was provided. Their study included all 65 American and Canadian medical universities; 95% of them received administrative help. To ensure effective quality control and develop a programme for risk management, the authors proposed the following measures: active support by the dean, setting goals and visions, appointing trained board members, developing tools for quality control, adhering to institutional standards in patient care, and establishing measures for continuous improvement [10]. 

The costs for learner-oriented quality improvement in advanced training (LQW) were ranked by size of the organisation and amounted to 4200 € for an organisation employing up to five persons, for example [http://www.elearning-mv.de/wp-content/uploads/2011/12/EmpfehlungenZertifizierungE-Learning-in-MV.pdf, cited 2014 September 10]. Costs for certification of correspondence courses quoted by the ZFU were reported to be at least 950 € or 150% of the purchasing price [http://www.zfu.de, cited 2014 September 10]. Thus, large sums of money have to be invested for the QMS before its advantages will pay off. According to the literature, use of a QMS contributes to cost reduction by applying standards and the option of re-using the system. Moreover, work processes can be streamlined, and staff costs may be lowered. In his study, Rothlauf concluded that the advantages associated with the implementation of a QMS justify the financial expenditure [16]. As reported by Knispel, cost reduction is a positive result of using a QMS [8]. However, high certification costs associated with implementing a QMS, as in the case of DIN ISO 9001, discourage small institutions from using a QMS, although cost reduction is mentioned as an advantageous feature of DIN ISO 9001 [17].

## Outlook

Our findings indicate, again, that the recommendation of the German Council of Science of July 04, 2008, to establish a QMS compliant with international standards at universities within a period of approximately three to five years [1] has not yet been realised, although there are several international initiatives that could serve as guidance. For example, the Distance Learning Accreditation in Europe (DLAE) project is a proposal for a European accreditation system in eLearning and blended learning. The criteria of the European system place more emphasis on educational methods than on administrative matters [2], [http://www.detc.org, cited 2014 September 10] compared to the American Distance Education and Training Council (DETC). The above-mentioned examples reflect the availability of a multitude of approaches for quality control in eLearning on both the national and international levels, indicating that the striving for quality in the context of eLearning concepts at universities is of considerable importance. In view of the choice of options and existing systems, universities are faced with the task to find a system that suits their individual needs or, alternatively, to consider adapting an existing quality control approach or QMS to their special requirements (example: Charité Berlin, Qualitätssiegel eLearning; individual seal in the style of DIN-PAS 1032-1). 

Based on the current literature, the following aspects in connection with implementing a QMS should be critically evaluated:

1. Certification of a system allows comparability and encourages competition. With individually designed QMS, the criteria assessed are not readily seen, and comparability with other QMS is barely possible.
2. Use of established standards is of substantial importance in the context of cost-saving measures.
3. External controllers of QMS tend to be preferable to internal controllers because they are expected to be more objective and may give fresh impetus to the institution.

## Conclusions

This investigation documents that only very few german medical universities already utilize a QMS for learning. Overall, we conclude that there is a considerable need for better information on QMS for eLearning.

## Acknowledgements

We thank all participating universities and Dr. Silvia M. Rogers Weller, Ph.D. (Mediwrite GmbH, Basel, Switzerland) for her language assistance.

## Authors’ contributions

Authors JA, JM and SG were involved in the conception and all in the evaluation of the study. All authors approved the final version of the manuscript to be published. All authors have been responsible for redrafting and revising the intellectual content of this article. The corresponding author wrote the first draft, the others contributed equally to the paper.

## Competing interes

The autors declare that they have no competing interests.

## Supplementary Material

Questionnaire
