# Supplementary material for: Quality management of eLearning for medical education: current situation and outlook
Source: GMS Z Med Ausbild. 2015 May 13;32(2):Doc20. doi: 10.3205/zma000962 (PMC4446651; doi:10.3205/zma000962)
Supplement: Questionnaire [file ZMA-32-20-s-001.pdf]

Dear quality management delegates, dear colleagues

This questionnaire aims at analysing and comparing quality management systems (QMS) for eLearning in your specific field to get a general idea of the current situation of quality management (QM) in eLearning.

The data collected by means of this questionnaire will be handled strictly confidentially and will only be used for the purpose of the present evaluation.

1. General information about your institution

1.1 Federal state

1.2 University

1.3 Specialised field: medical dental

1.4 Other specialised field (please indicate):

1.5 Type of institution: governmental private

1.6 Other (please indicate):

2. General questions

2.1 Have you implemented any QMS at your institution?

- Yes
- No

2.2 If YES, which type?

- QMS for teaching
- general QMS
- QMS for electronic teaching

2.3 Have you implemented a QMS for eLearning at your institution?

- Yes
- No

2.4 If NO, for the following reasons:

- no time
- too costly
- no need / no interest
- insufficient information about QMS in eLearning
- eLearning is not / hardly used
- We could not yet decide on a QMS.

2.5 No, for other reasons:

If you have not implemented any QMS for eLearning at your institution, please tick “NO” and name the reasons, if applicable. Please send the form back to us.

At this point, we would like to thank you very much for your cooperation!

2.6 Which specific QMS for eLearning do you know?

- DIN PAS 1032-1 (1)
- TUD eLearning Label (2)
- Q.E.D. (3)
- QSel (4)

- CEL (5)
- WebKolleg NRW (6)

2.7 Other QMS?

2.8 Which QMS for eLearning has been introduced at your institution?

- DIN PAS 1032-1 (1)
- TUD eLearning Label (2)
- Q.E.D. (3)
- QSel (4)
- CEL (5)
- WebKolleg NRW (6)

2.9 Other QMS?

2.10 Since when has the QMS been operational at your institution?

- 2001-2003
- 2004-2007

2.11 Remarks:

---

(1) DIN PAS 1032-1 Deutsches Institut für Normung e.V., Publicly Available Specification

(2) TUD eLearning Label Technische Universität Darmstadt

(3) Q.E.D. Qualitätsinitiative eLearning Deutschland

(4) QSel Qualitätssiegel eLearning

(5) CEL Certification of eLearning

(6) WebKolleg NRWNordrhein-Westfalen

---

3. Decision making

3.1 Why have you decided on the introduction of a QMS for eLearning?

- cost effectiveness / reduction of costs
- competition with other universities
- improved choice of eLearning programmes
- streamlining of processes

3.2 Other (please indicate):

3.3 Why have you chosen this particular QMS for eLearning?

- cost factor
- recommendation
- competition
- time factor
- competent service provider
- structural suitability for the university

3.4 Other (please indicate):

3.5 Would you choose the same QMS for eLearning again?

- Yes
- No

3.6 If NO, for the following reason:

- costs higher than expected
- time expenditure greater than expected
- not satisfied with the service provider
- individual adaptation to specific requirements not or hardly possible

3.7 Other reasons:

3.8 Which other QMS for eLearning has been discussed before implementing the chosen system?

3.9 Why have you decided against that QMS for eLearning?

3.10 Were there training sessions before the implementation of the QMS for eLearning?

- No
- Yes

3.11 If YES, what type of training?

4. Satisfaction, clarity

4.1 How satisfied are you with the chosen QMS for eLearning in general?

- very satisfied (1)
- mostly satisfied (2)
- moderately satisfied (3)
- hardly satisfied (4)
- not at all satisfied (5)

4.2 How would you rate the clarity of the chosen QMS for eLearning?

- very good (1)
- good (2)
- moderate (3)
- poor (4)
- very poor (5)

4.3 How would you rate the effectiveness of the QMS for eLearning in your special field?

- very high (1)
- high (2)
- moderate (3)
- low (4)
- very low (5)

4.4 Which positive effects have resulted from the implementation of the QMS for eLearning?

- none
- economic efficiency
- quality improvement
- competitiveness
- streamlined processes
- better choice of eLearning programmes
- improved learning outcomes
- reduced error rate

4.5 Other positive effects:

5. Time factor

5.1 How long did it take to implement the QMS for eLearning? (in months)

5.2 How much time is required for the maintenance of this QMS? (in hours per week, for all QM staff together)

5.3 Do you consider the time expenditure for QMS implementation acceptable?

- highly acceptable (1)
- acceptable (2)
- intermediate (3)
- not really acceptable (4)
- unacceptable (5)

5.4 Do you find the time expenditure for maintaining the QMS acceptable?

- highly acceptable (1)
- acceptable (2)
- intermediate (3)
- not really acceptable (4)
- unacceptable (5)

6. Cost factor

6.1 What were the costs for the implementation of the QMS for eLearning? (total cost and costs for certification in €)

6.2 What are the costs for maintaining the QMS? (€ per month)

6.3 Do you consider the costs for the implementation of the QMS for eLearning acceptable?

- highly acceptable (1)
- acceptable (2)
- intermediate (3)
- not really acceptable (4)
- unacceptable (5)

6.4 Do you find the costs for maintaining the QMS for eLearning acceptable?

- highly acceptable (1)
- acceptable (2)
- intermediate (3)
- not really acceptable (4)
- unacceptable (5)

7. Headcount and responsibilities

7.1 How many employees were required for setting up the QM system for eLearning?

- 1-5
- 5-10

7.2 If more than 10: how many?

7.3 How many employees are required for maintaining the QMS for eLearning?

- 1-5
- 5-10

7.4 If more than 10: how many?

7.5 Who is responsible for the QM system for eLearning?

- doctor/dentist
- faculty dean
- trained QM delegate

7.6 Other responsible person for QMS for eLearning:

8. General comments concerning QMS for eLearning

8.1 (blank)

Many thanks for your help!

8.2 The first 10 questionnaires returned to us will enter a prize draw for flash cards. Would you like to join the prize draw?

- No
- Yes, please keep me informed at the following e-mail address:

8.3 E-mail address
